# ﻿Taxonomy and phylogeny of the *Pleurotus djamor* complex with descriptions of a new species from China

**DOI:** 10.3897/mycokeys.126.162530

**Published:** 2025-12-10

**Authors:** Long Zeng, Yi-Hua Xu, Le-Le Wan, Yi-Fei Sun, Bao-Kai Cui

**Affiliations:** 1 State Key Laboratory of Efficient Production of Forest Resources, School of Ecology and Nature Conservation, Beijing Forestry University, Beijing 100083, China Beijing Forestry University Beijing China

**Keywords:** Basidiomycota, macrofungi, molecular phylogeny, morphology, new species

## Abstract

The *Pleurotus djamor* complex is widely distributed in tropical and subtropical regions and is known for its more or less reddish pileus. Phylogenetic analyses of the *P. djamor* complex were carried out using multiple loci, including the internal transcribed spacer regions (ITS), the translation elongation factor 1-alpha gene (tef1α), and the second largest subunit of RNA polymerase II (rpb2). In this study, a new species of *Pleurotus*, *P. sinensis*, is described based on morphological characters and molecular evidence. *Pleurotus sinensis* is characterized by a pileus that is white to pinkish buff or flesh-pink, flabelliform to petaloid, with inflexed and sometimes wavy margins. An illustrated description of the novel species is provided.

## ﻿Introduction

The genus *Pleurotus* (Fr.) P. Kumm. has high species diversity and a wide distribution around the world, and most species in this genus have notable edibility and medicinal value (Singer 1949; Guzmán 2000; Ma et al. 2015; Corrêa et al. 2016). However, the utilization and cultivation of *Pleurotus* have been constrained by misidentification and confused nomenclature (Vilgalys and Sun 1994; Zervakis et al. 2001).

*Pleurotus djamor* (Rumph. ex Fr.) Boedijn was first described by Rumphius (1750) as *Boletus fecundus arboreus*, and it was described as having an imbricate, circular-oblong to liver-shaped pileus displaying reddish coloration when young, fading to white, dull yellow, or yellowish tan at maturity, with a stipe that is short or sometimes lacking. Boedijn (1959) classified this species into the genus *Pleurotus*, and this classification has been followed ever since. Corner (1981) examined specimens of *P. djamor* in Malaysia based on morphological characteristics and habits and proposed six varieties, but these varieties were regarded as synonyms of *P. djamor*. Nevertheless, due to phenotypic plasticity and interfertility among species, morphology and sexual compatibility alone are insufficient for accurate species delimitation within the *P. djamor* complex (Nicholl and Petersen 2000). Specimens of *P. djamor* distributed in Mexico (Huerta et al. 2010), Brazil (Menolli Jr et al. 2014), and Kenya (Otieno et al. 2015) were clarified using ITS sequences. Zervakis et al. (2019) revealed high ITS heterogeneity among specimens of the *P. djamor* complex, and some materials labeled as *P. flabellatus* Sacc., *P. ostreatoroseus* Singer, and *P. salmoneostramineus* Lj.N. Vassiljeva also clustered into the same phylogenetic clade as the *P. djamor* complex. However, a comprehensive phylogenetic revision incorporating both morphological and multi-locus evidence remains lacking.

To resolve these taxonomic ambiguities, we conducted multi-locus analyses based on DNA sequences from the ITS, *tef1α*, and *rpb2* loci. In combination with morphological characteristics, these analyses led to the discovery of a new species within the *P. djamor* complex.

## ﻿Materials and methods

### ﻿Morphological studies

Specimens examined in this study were deposited at the herbarium of the Institute of Microbiology, Beijing Forestry University, China (BJFC). The morphological observation followed the protocols used in Lechner et al. (2004) and Camacho et al. (2012). Macro-morphological characteristics and habitats were documented based on field notes and laboratory observations. Special color terms followed Petersen (1996). Examination of the sections was conducted using a Nikon E80i microscope with phase-contrast illumination, capable of magnifications up to 1000× and manufactured by the Nikon Corporation in Tokyo, Japan. The observations, measurements, and illustrations were derived from slide preparations of dried material stained with Cotton Blue, 2% phloxine B and Melzer's reagent (Sun et al. 2022). Basidiospore measurements were taken from at least 30 spores per specimen, excluding 5% of extreme values, which are given in parentheses. The abbreviations used in this paper are as follows: IKIIKI = Melzer’s reagent, IKI− = the absence of dextrinoid or amyloid properties, KOH = 5% solution of potassium hydroxide, CB = Cotton Blue, CB+ = the cyanophilous reaction, CB− = the acyanophilous reaction, L = the mean length of spores (calculated as the arithmetic average), W = the mean width of spores (calculated as the arithmetic average), Q = the variability in the L/W ratio among the samples, and n (a/b) = the total number of spores (specified quantity/number of specimens).

### ﻿DNA extraction, PCR amplification, and sequencing

A CTAB plant genome rapid extraction kit-DN14 (Aidlab Biotechnologies Co., Ltd.) was employed for DNA extraction from dried specimens. The polymerase chain reaction (PCR) was conducted following the manufacturer’s guidelines with some modifications (Li et al. 2017; Zervakis et al. 2019).

The sequences of three gene loci were derived from PCR amplification. The internal transcribed spacer (ITS) regions were amplified with primer pairs ITS5 and ITS4 (White et al. 1990). The translation elongation factor 1-alpha gene (*tef1α*) was amplified with primer pairs EF1-983F and EF1-1567R (Rehner 2001). The second largest subunit of RNA polymerase II (rpb2) was amplified with primer pairs RPB2-3.1F and RPB2-6R2 (Liu and Hall 1999). The final PCR volume was 30 µL, consisting of 1 µL of each primer, 1 µL of extracted DNA, 12 µL of ddH_2_O, and 15 µL of 2× EasyTaq PCR Supermix (TransGen Biotech Co., Ltd., Beijing, China). PCR amplification was performed on an S1000™ Thermal Cycler (Bio-Rad Laboratories, CA, USA). The PCR procedure for ITS was as follows: initial denaturation at 94 °C for 4 min, followed by 35 cycles of denaturation at 94 °C for 40 s, annealing at 52 °C for 40 s, and extension at 72 °C for 1 min, with a final extension at 72 °C for 7 min. The PCR process for *rpb2* and *tef1α* was as follows: initial denaturation at 94 °C for 4 min, followed by 35 cycles at 94 °C for 40 s, 55 °C for 1 min, and 72 °C for 90 s, with a final extension at 72 °C for 10 min. The PCR products were subsequently purified and sequenced at the Beijing Genomics Institute (BGI, China) using the same primers. The newly generated and downloaded sequences in this study were deposited at GenBank and are listed in Table 1.

**Table 1.** Specimen information and GenBank accession numbers for the sequences used in this study.

Species name	Geographic Origin	Voucher	GenBank accession numbers			Reference
			ITS	*tef1*	*rpb2*	
*P. abieticola*	China	HKAS91342	KX836361	KX840302	KX870442	Li et al. (2017)
*P. abieticola*	China	HKAS45720	KP771696	KX885093	KX885220	Liu et al. (2015); Li et al. (2017)
*P. agave*	Mexico	CP-194	GU722262	–	–	Huerta et al. (2010)
*P. agave*	Mexico	ECS-0165	GU722264	–	–	Huerta et al. (2010)
*P. agave* (Submitted to NCBI under the name *P. opuntiae*)	Mexico	ET3313	AY450339	–	–	From NCBI
*P. agave* (Submitted to NCBI under the name *P. opuntiae*)	Mexico	TENN52368	AY450340	–	–	From NCBI
*P. calyptratus*	Austria	TENN57451	AY450338	–	–	From NCBI
*P. calyptratus*	Slovakia	CBS 325.85	EU424283	–	–	From NCBI
*P. calyptratus*	Russia	1935	KF932720	–	–	Shnyreva and Shnyreva (2015)
*P. djamor*	Cuba	CBS596.96	FJ040176	–	–	From NCBI
*P. djamor*	Mexico	ECS-0123	GU722265	–	–	Huerta et al. (2010)
*P. djamor*	Mexico	ECS-0128	GU722266	–	–	Huerta et al. (2010)
*P. djamor*	Mexico	ECS-0130	GU722267	–	–	Huerta et al. (2010)
*P. djamor*	Mexico	ECS-0150	GU722268	–	–	Huerta et al. (2010)
*P. djamor*	Mexico	ECS-0151	GU722269	–	–	Huerta et al. (2010)
*P. djamor*	Mexico	ECS-0159	GU722273	–	–	Huerta et al. (2010)
*P. djamor*	Mexico	CP-170	GU722271	–	–	Huerta et al. (2010)
*P. djamor*	Brazil	SP445682	KF280324	–	–	Menolli Jr et al. (2014)
*P. djamor*	Brazil	SP445798	KF280326	–	–	Menolli Jr et al. (2014)
*P. djamor*	Dominican Republic	TENN F-59778	KP026246	–	–	From NCBI
*P. djamor*	Mexico	CC050	KX573921	–	–	Aguilar Doroteo et al. 2018
*P. djamor*	Mexico	CC051	KX573922	–	–	Aguilar Doroteo et al. 2018
*P. djamor*	Mexico	CC053	KX573924	–	–	Aguilar Doroteo et al. 2018
*P. djamor*	Mexico	CC056	KX573927	–	–	Aguilar Doroteo et al. 2018
*P. djamor*	Sri Lanka	HKAS94069	KX061789	KX840308	–	Li et al. (2017)
*P. djamor* (Submitted to NCBI under the name *P. ostreatoroseus*)	unknown	P94	MG282434	–	–	From NCBI
*P. djamor* (Submitted to NCBI under the name *P. ostreatoroseus*)	Peru	ITA-308	ON426447	–	–	From NCBI
*P. djamor*	Puerto Rico	Cui 16861	PV771009	PX608114	PX608104	This study
*P. djamor*	Puerto Rico	Cui 16862	PV771010	PX608115	PX608105	This study
*P. djamor*	Puerto Rico	Cui 16890	PV771011	PX608116	PX608106	This study
*P. djamor*	Puerto Rico	Cui 16902	PV771012	PX608117	PX608107	This study
P. djamor var. fuscopruinosus (Submitted to NCBI under the name *P. salmoneostramineus*)	unknown	ACCC50836	EU424302	–	–	From NCBI
P. djamor var. fuscopruinosus (Submitted to NCBI under the name P. djamor var. roseus)	unknown	ABM1049204	KC582640	–	–	From NCBI
P. djamor var. fuscopruinosus (Submitted to NCBI under the name *P. flabellatus*)	Malaysia	ATCC38137	AY265827	–	–	From NCBI
P. djamor var. fuscopruinosus (Submitted to NCBI under the name *P. flabellatus*)	unknown	ATCC38140	AY368660	–	–	From NCBI
P. djamor var. fuscopruinosus (Submitted to NCBI under the name *P. djamor*)	Malaysia	FUM-093	KY951475	–	–	Avin et al. (2017)
P. djamor var. fuscopruinosus	Thailand	MFLUCC24-0062	PP192013	–	–	Phonemany et al. (2025)
*Pleurotus* sp. 1 (Submitted to NCBI under the name *P. opuntiae*)	New Zealand	ICMP 11566	MH395961	–	–	From NCBI
*Pleurotus* sp. 1 (Submitted to NCBI under the name *P. opuntiae*)	New Zealand	ICMP 11670	MH395966	–	–	From NCBI
*Pleurotus* sp. 1 (Submitted to NCBI under the name *P. opuntiae*)	New Zealand	ICMP 11671	MH395967	–	–	From NCBI
*Pleurotus* sp. 1 (Submitted to NCBI under the name *P. parsonsiae*)	New Zealand	ICMP 18169	MH395975	–	–	From NCBI
*Pleurotus* sp. 2 (Submitted to NCBI under the name *P. djamor*)	Nigeria	ELEB27	KT273359	–	–	Adedokun et al. (2016)
*Pleurotus* sp. 2 (Submitted to NCBI under the name *P. djamor*)	Kenya	KKF8428	KJ754106	–	–	Otieno et al. (2015)
*Pleurotus* sp. 2 (Submitted to NCBI under the name *P. djamor*)	Kenya	KKF2710	KJ754108	–	–	Otieno et al. (2015)
*Pleurotus* sp. 2 (Submitted to NCBI under the name *P. djamor*)	Kenya	KKF0121	KJ754109	–	–	Otieno et al. (2015)
*P. opuntiae*	Italy	SAF 250	MH620770	–	–	Zervakis et al. (2019)
*P. opuntiae*	Italy	SAF 251	MH620771	–	–	Zervakis et al. (2019)
*P. opuntiae*	Italy	SAF 252	MH620772	–	–	Zervakis et al. (2019)
*P. sinensis*	China	HKAS90179	KX836373	KX840306	KX870451	Li et al. (2017)
*P. sinensis*	China	HKAS90178	KX836374	–	KX870452	Li et al. (2017)
*P. sinensis* (Submitted to NCBI under the name *P. salmoneostramineus*)	unknown	ASI 2172	AY265845	–	–	From NCBI
*P. sinensis*	China	Dai 16572	PX612360	PX608118	PX608108	This study
*P. sinensis*	China	Cui 23262	PV771017	PX608119	PX608109	This study
*P. sinensis*	China	Cui 23263	PV771018	PX608120	PX608110	This study
*P. sinensis*	China	Cui 23999	PX612361	PX608123	PX608113	This study
*P. sinensis*	China	Cui 23439	PV771021	PX608121	PX608111	This study
*P. sinensis*	China	Cui 23450	PV771022	PX608122	PX608112	This study

### ﻿Phylogenetic analyses

In this study, 87 sequences derived from 59 fungal samples representing nine species were used to reconstruct the phylogenetic trees, including 59 ITS sequences, 14 *tef1α* sequences, and 14 *rpb2* sequences. Among them, 30 sequences were newly generated, comprising 10 ITS sequences, 10 *tef1α* sequences, and 10 *rpb2* sequences.

The phylogenetic relationships within the *P. djamor* complex were inferred using ITS and the combined three-gene (ITS + *tef1α* + *rpb2*) datasets. The ITS dataset was used to reconstruct the general phylogenetic framework of the complex, whereas the combined three-gene datasets were used to further determine the phylogenetic differences between *P. sinensis* and *P. djamor*. *Pleurotus abieticola* R.H. Petersen & K.W. Hughes was selected as the outgroup. The datasets were aligned in MAFFT 7 (Katoh and Standley 2013; https://mafft.cbrc.jp/alignment/server/) and manually adjusted in BioEdit (Hall 1999). Alignment statistics, including the numbers of constant, parsimony-uninformative, and parsimony-informative sites, were calculated in PAUP version 4.0b10 (Swofford 2002) to evaluate the phylogenetic information content of the datasets. Alignments were spliced in Mesquite v. 3.2 (Maddison and Maddison 2017). Phylogenetic analyses were performed using the maximum likelihood (ML) and Bayesian inference (BI) methods. ML analyses were performed using RAxML-HPC v. 8.2.3 (Stamatakis 2014) with 1000 ML searches under the GTRGAMMA model, and only the maximum likelihood best tree from all searches was kept. In addition, 1000 rapid bootstrap replicates were run with the GTRCAT model to assess ML bootstrap values. BI analyses were performed using MrBayes v. 3.2 (Ronquist and Huelsenbeck 2003) with two simultaneous independent chains for datasets, performing 10 million generations until the split deviation frequency value was < 0.01 and sampled every 100 generations. The first 25% of sampled trees were discarded as burn-in, while the remaining ones were used to calculate Bayesian posterior probabilities (BPP) of the clades. The ML bootstrap (ML-BS) ≥ 70% and Bayesian posterior probabilities (BPP) ≥ 0.90 were presented on topologies from the ML analyses. The trees were viewed in FigTree v. 1.4.3 (http://tree.bio.ed.ac.uk/software/figtree/).

## ﻿Results

### ﻿Phylogenetic analyses

**Figure 1.**
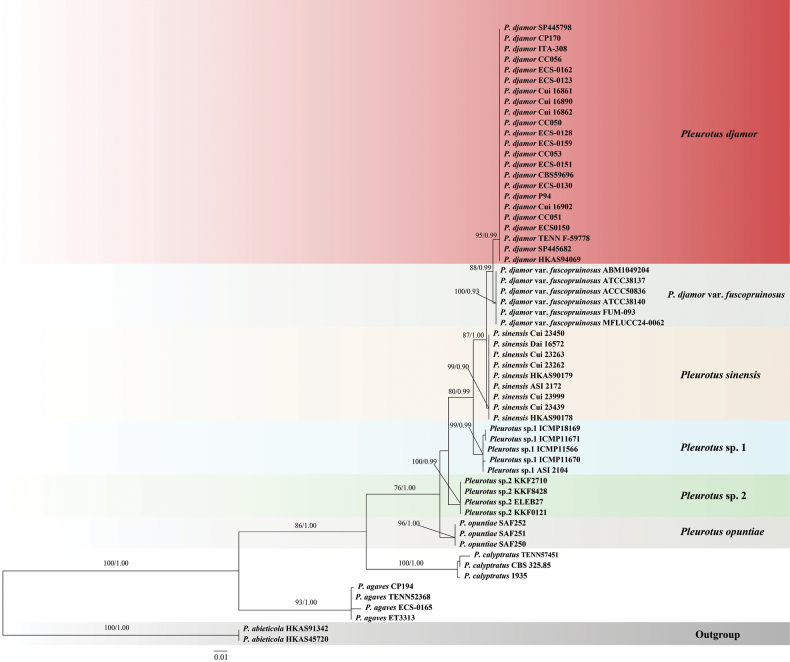
Phylogenetic analysis of the *Pleurotus djamor* complex based on the ITS sequence dataset.

The ITS dataset included sequences from 59 fungal samples representing nine taxa. The dataset had an aligned length of 647 characters, including 467 constant characters, three parsimony-uninformative variables, and 177 parsimony-informative characters. Bayesian and ML analyses resulted in the same topology. Only the ML tree is provided in Fig. 1, and the ML bootstrap (ML-BS) and Bayesian posterior probabilities (BPP) are shown at the nodes. The phylogenetic tree showed that the species originally labeled as *Pleurotus djamor* were divided into five distinct monophyletic clades (Fig. 1): *P. djamor* from Sri Lanka and the Americas (95% ML-BS, 0.99 BPP), P. djamor var. fuscopruinosus from Thailand and Malaysia (100% ML-BS, 0.93 BPP), *Pleurotus sinensis* sp. nov. from China (99% ML-BS, 0.90 BPP), *Pleurotus* sp. 1 from New Zealand (99% ML-BS, 0.99 BPP), and *Pleurotus* sp. 2 from Africa (100% ML-BS, 1.00 BPP).

**Figure 2.**
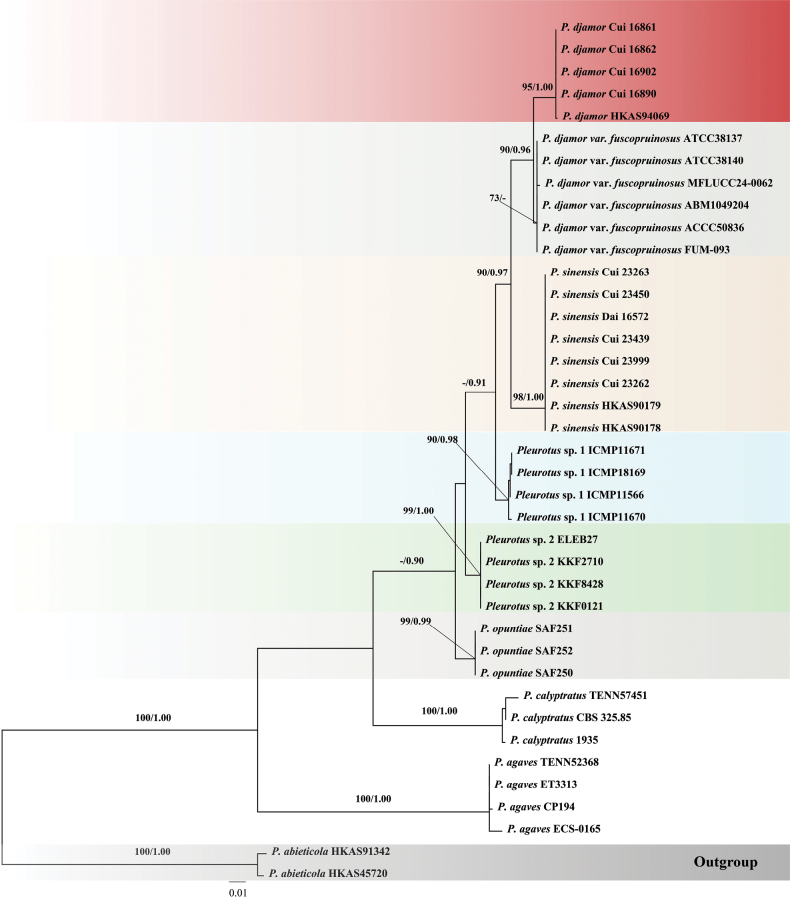
Phylogenetic analysis of the *Pleurotus djamor* complex based on the combined ITS + *tef1α* + *rpb2* dataset.

The combined three-gene (ITS + *tef1α* + *rpb2*) dataset included sequences from 39 fungal samples representing nine taxa. The dataset had an aligned length of 1989 bp, including 1474 constant characters, 34 parsimony-uninformative variables, and 481 parsimony-informative characters. Bayesian and ML analyses resulted in the same topology. Only the ML tree is provided in Fig. 2, and the ML bootstrap (ML-BS) and Bayesian posterior probabilities (BPP) are shown at the nodes. The phylogenetic tree showed that *P. djamor* and *P. sinensis* clustered into two distinct groups with high support (95% ML-BS, 1.00 BPP for *P. djamor*; 98% ML-BS, 1.00 BPP for *P. sinensis*; Fig. 2).

### ﻿Taxonomy

#### ﻿ Pleurotus sinensis

Taxon classificationFungiAgaricalesPleurotaceae

L. Zeng, Y.F. Sun & B.K. Cui sp. nov.

502D2EC4-654B-576E-A0C4-7952CDDA9A94

861390

Figs 3, 4

##### Diagnosis.

*Pleurotus sinensis* is characterized by a pileus that is white to pinkish buff or flesh-pink pileus when young, turning yellow at maturity, flabelliform, spatulate, or petaloid in shape, with inflexed and occasionally wavy margins with age.

##### Holotype.

China • Liaoning Province, Shenyang, Dadong District, 3 July 2024, Cui 23439 (BJFC).

##### Etymology.

“*sinensis*” (Lat.) refers to specimens derived from China.

##### Description.

***Pileus*** 30–70 × 35–90 mm, flabelliform, spatulate, or petaloid, white to pinkish buff (5A3) or flesh-pink (8A3/9A4) when young, becoming yellow at maturity; with inflexed and wavy margin with age; glabrous, smooth to touch; margin entire (Fig. 4). ***Lamellae*** decurrent, margin entire, l.5–3 mm in dry state, white when young becoming pinkish buff (5A3) when old. ***Stipe*** 5–15 mm long × 5–10 mm diam, laterally stipitate or sessile. ***Context*** 1–1.5 mm thick when dry.

**Figure 3.**
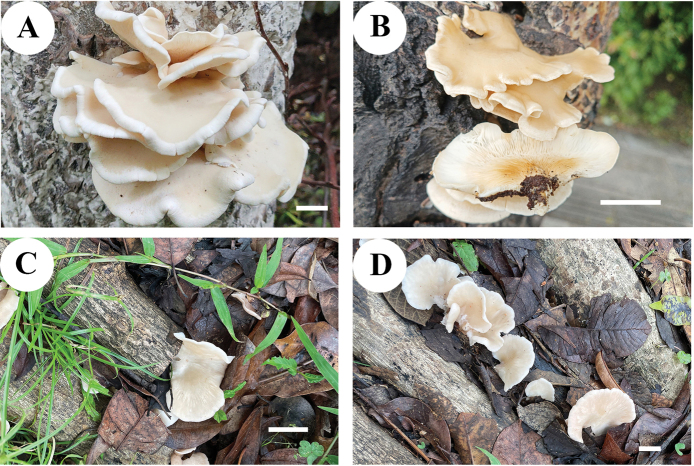
Basidiomata of *Pleurotus sinensis* in the field. **A.** (Cui 23439, holotype); **B.** (Cui 23450, paratype); **C.** (Cui 23262, paratype); **D.** (Cui 23263, paratype). Scale bars: 1 cm.

**Figure 4.**
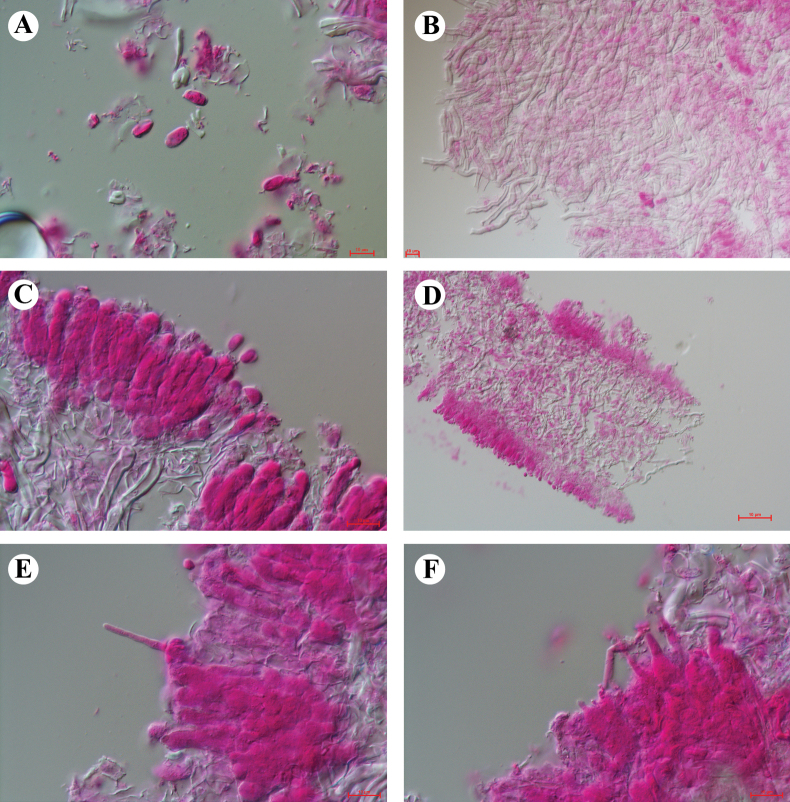
Microscopic structures of *Pleurotus sinensis* (Cui 23439, holotype). **A.** Basidiospores; **B.** Hyphae of the hymenium; **C.** Basidia and basidioles; **D.** Lamella section; **E, F.** Cheilocystidia. Scale bars: 10 µm (**A–F**).

***Basidiospores*** (6.2–) 6.8–9.8 (–10.2) × (3.3–) 3.5–5.5 (–5.7) μm, L = 8.08 μm, W = 4.18 μm, Q = 1.78–2.11 (n = 60/2), cylindrical-oblong, hyaline, thin-walled, smooth, IKI–, CB–. ***Basidia*** 23.6–28.3 × 5.2–6.8 μm, clavate, 4–spored, hyaline, thin-walled. ***Basidioles*** 15.8–24.2 × 4.9–5.2 μm, in shape similar to basidia. ***Cheilocystidia*** hyaline, thin-walled, clavate with mucronate, 22–41.7 × 2.6–3.4 μm. ***Pleurocystidia*** absent. ***Hymenophoral trama*** dimitic, with clamped generative hyphae 3.6–6.8 μm diam and skeletal hyphae 2.5–4.9 μm diam. ***Pileus trama*** dimitic, with generative hyphae 3.4–7.7 μm diam and skeletal hyphae 2.5–4.8 μm diam. ***Stem context*** also dimitic, with generative hyphae 4.5–6.8 μm diam and skeletal 2.6–5.2 μm diam.

##### Habitat and distribution.

Solitary, gregarious to imbricate, on angiosperm trees or on dead and decaying wood in the subtropical and temperate zones of China.

##### Additional specimens examined (paratypes).

China • Guizhou Province, Guiyang, Guizhou Academy of Agricultural Sciences, 17 June 2016, Dai 16572 (BJFC); • Guangdong Province, Shenzhen, Futian District, Futian Mangrove Ecological Park, 20 April 2024, Cui 23262 (BJFC), Cui 23263 (BJFC); • Sichuan Province, Chengdu, 1 August 2024, Cui 23999 (BJFC); • Liaoning Province, Fushun, 4 July 2024, Cui 23450 (BJFC).

#### ﻿ Pleurotus djamor

Taxon classificationFungiAgaricalesPleurotaceae

(Rumph. ex Fr.) Boedijn, Rumphius Mem. Vol. 292, 1959.

9FA0E985-09D7-5882-A9BD-03D856FF0567

355683

Figs 5, 6

##### Diagnosis.

*Pleurotus djamor* is characterized by a pileus that is clay-pink to salmon or white when young, becoming thicker and turning brown at maturity.

##### Description.

***Pileus*** 20–70 × 20–100 mm, flabelliform, clay-buff to salmon (6B/C4–6A4) or white when young, brown at maturity; surface dry, glabrous; margin entire. ***Lamellae*** decurrent, margin entire, l–7.5 mm in dry state, white when young becoming pinkish buff (5A3) when old. ***Stipe*** 5–10 mm long, 4–8 mm in diam., laterally stipitate or sessile. ***Context*** 1–3 mm thick when dry.

***Basidiospores*** (6.8–) 7.0–9.5 (–10.6) × (2.8–) 3.1–4.2 (–4.4) μm, L = 8.0 μm, W = 3.54 μm, Q = 2.24–2.28 (n = 60/2), cylindrical-oblong, hyaline, thin-walled, smooth, IKI–, CB–. ***Basidia*** 27.1–31.8 × 5.2–7.3 μm, clavate, 4–spored, hyaline, thin-walled. ***Basidioles*** 23.1–27.7 × 5.8–6.6 μm, in shape similar to basidia. ***Cheilocystidia*** hyaline, thin-walled, clavate with mucronate, 27.9–32.3 × 6.3–7.9 μm. ***Pleurocystidia*** absent. ***Hymenophoral trama*** dimitic, with clamped generative hyphae 3.7–8.7 μm in diam, and skeletal hyphae 2.9–5.8 μm in diam. ***Pileus trama*** dimitic, with generative hyphae 4.0–8.5 μm in diam, and skeletal hyphae 3.1–5.2 μm in diam. ***Stem context*** dimitic, with generative hyphae 3.4–6.8 μm in diam, and skeletal hyphae 2.6–4.2 μm in diam.

**Figure 5.**
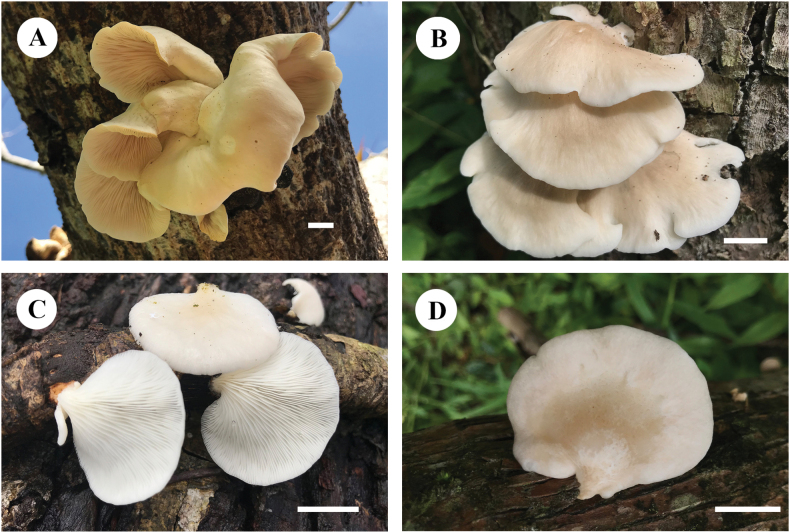
Basidiomata of *Pleurotus djamor* in the field. **A.** (Cui 16861); **B.** (Cui 16862); **C.** (Cui 16902); **D.** (Cui 16890). Scale bars: 1 cm.

**Figure 6.**
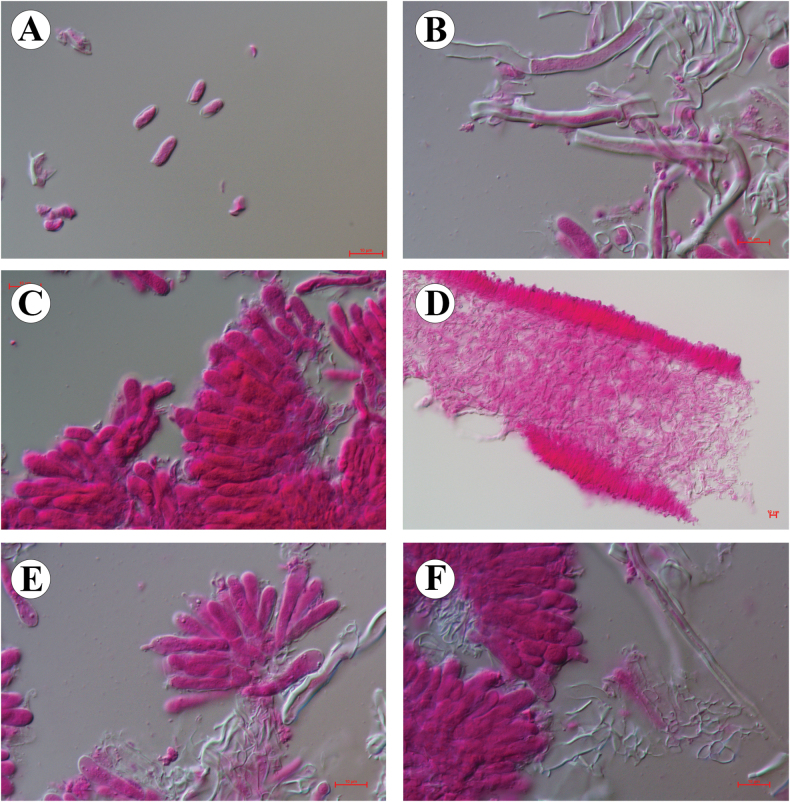
Microscopic structures of *Pleurotus djamor* (Cui 16861). **A.** Basidiospores; **B.** Hyphae of the hymenium; **C.** Basidia and basidioles; **D.** Lamella section; **E, F.** Cheilocystidia. Scale bars: 10 µm (**A–F**).

##### Habitat and distribution.

Solitary, gregarious to imbricate, on angiosperm trees in the tropical forests of Southeast Asia and the Americas.

##### Specimens examined in this study.

Puerto Rico • San Juan, 15 July 2018, Cui 16855 (BJFC); • Rio Abajo State Forest Park, 16 July 2018, Cui 16861 (BJFC) & Cui 16862 (BJFC); • Carite State Forest Park, 19 July 2018, Cui 16890 (BJFC) & Cui 16902 (BJFC) & Cui 16920 (BJFC) & Cui 16922 (BJFC) & Cui 16925 (BJFC).

## ﻿Discussion

In this study, the ITS phylogenetic tree revealed that the *P. djamor* complex was divided into five monophyletic clades (Fig. 1). *Pleurotus djamor* consists of specimens originating from Sri Lanka and America, which can be identified as authentic *P. djamor* based on their morphology. Closely related to *P. djamor* is its sister group, P. djamor var. fuscopruinosus, from Thailand and Malaysia. *Pleurotus sinensis*, newly described from China in this study, is closely related to *P. djamor* and P. djamor var. fuscopruinosus in the phylogenetic tree. *Pleurotus* sp. 1 and *Pleurotus* sp. 2 were derived from New Zealand and Africa, respectively. They formed distinct and stable clades in the phylogenetic tree and were distinct from other taxa within the *P. djamor* complex. However, their taxonomic status requires further clarification in future studies due to the lack of specimens from the corresponding regions. *Pleurotus opuntiae* (Durieu & Lev.) Sacc. was confirmed as an independent species by Zervakis et al. (2019), and it is currently known to be distributed only in the Mediterranean region. It is noteworthy that several ITS sequences within this complex were originally labeled under different names: P. djamor var. roseus (KC582640), *P. flabellatus* (AY265827, AY368660), *P. opuntiae* (MH395961, MH395966, MH395967), *P. ostreatoroseus* (MG282434, ON426447), *P. parsonsiae* (MH395975), and *P. salmoneostramineus* (AY265845, EU424302). P. djamor var. roseus, *P. flabellatus*, *P. ostreatoroseus*, and *P. salmoneostramineus* have previously been regarded as synonyms of *P. djamor* (Corner 1981; Nicholl and Petersen 2000; Zervakis et al. 2019). Nevertheless, in this study, the sequences labeled as *P. ostreatoroseus* were placed within *P. djamor*; the sequences labeled as P. djamor var. roseus and *P. flabellatus* were placed within P. djamor var. fuscopruinosus; and the sequences labeled as *P. salmoneostramineus* were placed within three distinct clades. The sequences from New Zealand, originally labeled as *P. opuntiae* (MH395961, MH395966, MH395967) and *P. parsonsiae* (MH395975), are considered to represent a distinct phylogenetic species. To further distinguish *P. sinensis* from *P. djamor*, a combined three-gene (ITS + *tef1α* + *rpb2*) dataset was used. Phylogenetic analyses based on this dataset revealed a distinct grouping of *P. sinensis* specimens relative to *P. djamor*, with strong statistical support (Fig. 2).

Morphologically, *P. djamor* is characterized by a pileus that is clay-pink to salmon or white when young, becoming thicker and turning brown at maturity, and the shape of the pileus is flabelliform or spathulate.

Pleurotus djamor var. fuscopruinosus is characterized by a flabelliform greyish to white pileus, greyish fuliginous pruinosity at the base, and clavate cheilocystidia with brownish walls. The pileipellis hyphae exhibit encrustation and show minute fascicles at the end cells (Phonemany et al. 2025).

*Pleurotus sinensis* differs from *P. djamor* and P. djamor var. fuscopruinosus by its flabelliform, spatulate, or petaloid pileus that is white to pinkish buff or flesh-pink when young, turning yellow at maturity, with inflexed and occasionally wavy margins with age.

*Pleurotus opuntiae* differs from *P. djamor*, P. djamor var. fuscopruinosus, and *P. sinensis* by its pileus, which is whitish or grayish and then cream or beige; the shape is initially convex, becoming applanate and finally concave; and the surface is radially appressed fibrillose to squamulose (Zervakis et al. 2019).

The morphological differences between *P. djamor* and its related species are shown in Table 2.

**Table 2.** The distinctive discriminating morphological characters for the *Pleurotus djamor* species complex.

Source	Species or variety	Spore size (μm)		Q value	Cystidia	Hyphal structure
		Length	Width			
This study	*P. djamor*	7.0–9.5	3.1–4.2	2.44	Cheilocystidia Mucronate, 27.9–32.3 × 6.3–7.9 μm; Pleurocystidia absent	Dimitic
Phonemany et al. 2025	P. djamor var. fuscopruinosus	6.0–8.0	3.0–4.5	1.44–2.18	Cheilocystidia short clavate to clavate with thin walls and brownish coloration, 11.9–31.7 × 6.4–12.2 μm; Pleurocystidia absent	Dimitic
Zervakis et al. 2019	*P. opuntiae*	9.2–10.8	4.3–4.9	2–2.40	Cheilocystidia subcylindrical, subclavate, utriform or pyriform, 19–44 × 6–9.5 μm; Pleurocystidia absent	Dimitic
This study	*P. sinensis*	6.8–9.8	3.5–4.5	1.78–2.11	Cheilocystidia clavate with mucronate, 22–41.7 × 2.6–3.4 μm; Pleurocystidia absent	Dimitic

Although the *P. djamor* complex has been reported in many areas such as Asia, America, Oceania, and Africa, its taxonomic status and phylogenetic relationship remained ambiguous for a long time. Here we first performed phylogenetic analyses on the *P. djamor* complex based on multiple gene fragments, clarified the composition of this complex, and identified a new species. The identification results indicated that geographical isolation plays a crucial role in the genetic evolution of the *P. djamor* complex, despite previous studies showing no obvious morphological differences between specimens from different geographical regions of *P. djamor* (Nicholl and Petersen 2000). In the future, the taxonomic status of the unclassified clades will be clarified with additional specimen collections and molecular evidence.

## Supplementary Material

XML Treatment for Pleurotus sinensis

XML Treatment for Pleurotus djamor

## ﻿Additional information

### Use of AI

No use of AI was reported.

### Author ORCIDs

Long Zeng https://orcid.org/0009-0004-0124-459X

Yi-Hua Xu https://orcid.org/0009-0007-8997-2517

Le-Le Wan https://orcid.org/0009-0002-9625-0962

Yi-Fei Sun https://orcid.org/0000-0003-3997-3662

Bao-Kai Cui https://orcid.org/0000-0003-3059-9344

### Data availability

All data that support the findings of this study are available in the main text or in publicly accessible data repositories, as indicated in the text.
